# Efficacy and Safety of Upacicalcet in Hemodialysis Patients with Secondary Hyperparathyroidism: A Randomized Placebo-Controlled Trial

**DOI:** 10.2215/CJN.0000000000000253

**Published:** 2023-09-11

**Authors:** Takashi Shigematsu, Fumihiko Koiwa, Yoshitaka Isaka, Masafumi Fukagawa, Keiko Hagita, Yukihisa S. Watanabe, Daisuke Honda, Tadao Akizawa

**Affiliations:** 1 Division of Nephrology, Rinku General Medical Center, Osaka, Japan; 2 Division of Nephrology, Department of Internal Medicine, Showa University Fujigaoka Hospital, Yokohama, Japan; 3 Department of Nephrology, Osaka University Graduate School of Medicine, Osaka, Japan; 4 Division of Nephrology, Endocrinology, and Metabolism, Department of Internal Medicine, Tokai University School of Medicine, Kanagawa, Japan; 5 Clinical Development Department, Sanwa Kagaku Kenkyusho Co., Ltd., Nagoya, Japan; 6 Project Management Department, Sanwa Kagaku Kenkyusho Co., Ltd., Nagoya, Japan; 7 Division of Nephrology, Department of Medicine, Showa University School of Medicine, Tokyo, Japan

**Keywords:** calcium-sensing receptor, dialysis, mineral metabolism

## Abstract

**Background:**

Secondary hyperparathyroidism is a major complication of patients undergoing hemodialysis (HD). Upacicalcet, a new injectable calcimimetic, acts on calcium-sensing receptors to suppress parathyroid hormone (PTH) secretion. We examined the efficacy and safety of upacicalcet in patients with secondary hyperparathyroidism receiving HD.

**Methods:**

In this phase 3, double-blind, placebo-controlled study, we randomized Japanese patients undergoing HD with serum intact PTH (iPTH) concentrations >240 pg/ml and corrected calcium concentrations ≥8.4 mg/dl. Either upacicalcet or placebo was administered after each HD session for 24 weeks. The primary outcome was the percentage of participants achieving the target mean serum iPTH concentration (60–240 pg/ml) at weeks 22–24.

**Results:**

A total of 103 participants received upacicalcet, and 50 participants received the placebo. The percentage of participants achieving mean serum iPTH concentrations of 60–240 pg/ml during the evaluation period was 67% (69/103) in the upacicalcet group and 8% (4/50) in the placebo group. The difference between the two groups was 59% (95% confidence interval, 48% to 71%). Upacicalcet also decreased serum fibroblast growth factor-23, bone-specific alkaline phosphatase, total type 1 procollagen-N-propeptide, and tartrate-resistant acid phosphatase-5b concentrations. Adverse events were reported in 85% (88/103) and 72% (36/50) participants in the upacicalcet and placebo groups, respectively. The incidence of upper gastrointestinal adverse events, such as nausea and vomiting, was similar between the two groups. Serum corrected calcium concentrations <7.5 mg/dl were observed in 2% of participants in the upacicalcet group and no participants in the placebo group.

**Conclusions:**

Upacicalcet, a novel injectable calcimimetic, is effective and safe for secondary hyperparathyroidism patients receiving HD.

**Clinical Trial Registry Name and Registration Number:**

Phase 3 Study of SK-1403, NCT03801980.

## Visual Abstract

**Figure:**
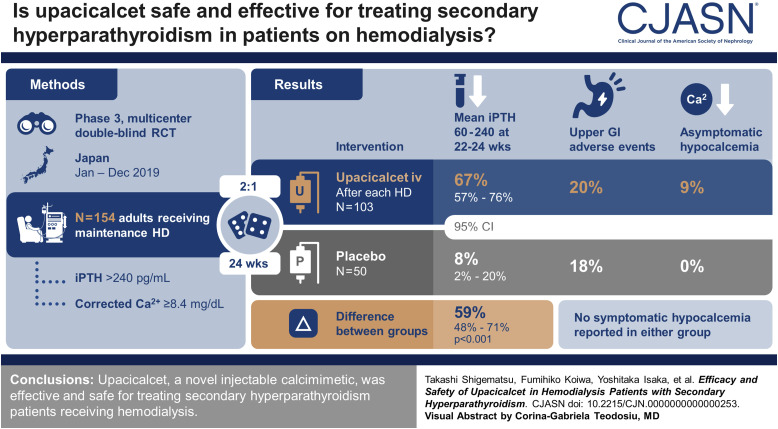

## Introduction

Secondary hyperparathyroidism is a major pathognomonic condition representative of Chronic Kidney Disease–Mineral and Bone Disorders (CKD–MBDs), particularly in patients undergoing hemodialysis (HD), and can lead to vascular calcification and osteitis fibrosa.^1–3^

Calcimimetics inhibit parathyroid hormone (PTH) production and secretion, playing a central role in secondary hyperparathyroidism management.^3,4^ Cinacalcet, a first-generation oral calcimimetic, reduces serum intact PTH (iPTH), Ca, and P concentrations.^5–7^ It may also decrease the risk of vascular calcification and mortality.^8,9^ However, cinacalcet causes gastrointestinal adverse events, including nausea and vomiting, resulting in low adherence.^10,11^ Furthermore, it should be administered with caution in patients receiving cytochrome P450 (CYP) 3A4 inhibitors or 2D6 substrates and patients with hepatic dysfunction.^12^ To overcome these challenges, evocalcet, another oral calcimimetic that does not inhibit CYP2D6 and is associated with a lower incidence of gastrointestinal adverse events, was approved in Japan.^13,14^ Furthermore, drug adherence improved after the advent of etelcalcetide, an injectable calcimimetic d-amino acid peptide.^15,16^ However, etelcalcetide is suggested to pose the highest risk of hypocalcemia among the calcimimetic agents.^16,17^ Moreover, contrary to expectations, etelcalcetide did not reduce gastrointestinal adverse events compared with oral cinacalcet.^16,17^ Therefore, calcimimetics with no adherence issues and low risk of upper gastrointestinal adverse events and hypocalcemia are required.

Upacicalcet is a novel injectable one–amino acid small-molecule calcimimetic derived from *γ*-glutamyl peptides.^18^ Nonclinical studies showed that upacicalcet was virtually unmetabolized in the liver and was unlikely to inhibit or induce CYP activity. Phase 1 and 2 clinical trials showed that its interdialytic plasma half-life was 65–122 hours in HD patients, which is markedly longer than that in healthy participants.^19,20^ Moreover, its removal rate in a HD session was 79%–100%. Therefore, the plasma concentration of upacicalcet plateaus after 1 week of administration after each dialysis session, contrary to etelcalcetide.^20^ These findings showed that <400 *µ*g of upacicalcet did not cause upper gastrointestinal adverse events. Upacicalcet having unique pharmacokinetic properties may be efficacious and not cause adverse events due to overexposure.

Consequently, we hypothesized that upacicalcet reduces serum iPTH concentrations while reducing the incidence of hypocalcemia and upper gastrointestinal adverse events. In this study, to examine the efficacy and safety of upacicalcet, we conducted a phase 3, multicenter, randomized, double-blind, placebo-controlled, parallel-group study.

## Methods

### Study Design

This phase 3, multicenter, randomized, double-blind, placebo-controlled, parallel-group study was conducted between January and December 2019 at 41 sites in Japan (Supplemental Table 1). The study design is illustrated in Supplemental Figure 1. We complied with the Declaration of Helsinki, Good Clinical Practice, and related laws and regulations. The study protocol was reviewed and approved by the Institutional Review Board of each study site. Written informed consent was obtained from all the participants. This study was registered in ClinicalTrials.gov (NCT03801980).

### Patients

The inclusion criteria were as follows: Japanese patients with CKD 20 years or older, receiving HD or hemodiafiltration three times a week for ≥12 weeks before screening, serum iPTH concentrations >240 pg/ml for two consecutive weeks, and serum corrected Ca for albumin concentrations ≥8.4 mg/dl during the screening period. The exclusion criteria are listed in Supplemental Document 1.

Enrolled participants were randomized to either the upacicalcet or placebo treatment groups in a ratio of 2:1. Dynamic allocation using the minimization method was performed. The allocation factors were serum iPTH concentrations (≥500 and <500 pg/ml) and serum corrected calcium (cCa) concentrations (≥9.0 and <9.0 mg/dl) at 1 week before treatment with study drugs (blinded upacicalcet or placebo). Vitamin D receptor activators (VDRAs) were not initiated or subjected to dosage change, except for rescue therapy, until the end of the study (Supplemental Figure 1). Dialysis conditions, including dialysate calcium concentrations, were not changed during the study period. The allocation was performed centrally and independently by EPS Corporation (Tokyo, Japan).

### Treatment

The study drug was administered three times a week into the venous side of the dialysis circuit during blood return at the end of HD. The treatment duration was 24 weeks. The initial dose was 50 *μ*g if the serum cCa concentrations were ≥9.0 mg/dl, and this dose was reduced to 25 *μ*g if serum cCa concentrations were lower (relatively hypocalcemic). The dose was adjusted in seven steps: 25, 50, 100, 150, 200, 250, and 300 *μ*g. During dose adjustment, the dose was increased stepwise. The criteria for increasing, decreasing, and interrupting study drug administration are shown in Supplemental Figure 1. Weeks 22–24 were defined as the evaluation period where dose titration was completed and fixed.

### Efficacy Assessments

The primary outcome was the percentage of participants achieving mean serum iPTH concentrations of 60–240 pg/ml according to the CKD–MBD guidelines of the Japanese Society for Dialysis Therapy (JSDT) during the evaluation period (weeks 22, 23, and 24).^21^ The secondary outcomes were the percentages of participants achieving ≥30% and ≥50% reductions in mean serum iPTH concentrations from baseline (weeks −2, −1, and 0) during the evaluation period. Furthermore, serum iPTH, cCa, and P concentrations were evaluated. Serum intact fibroblast growth factor-23 (FGF23), bone-specific alkaline phosphatase (BAP), total type 1 procollagen-N-propeptide (total P1NP), and tartrate-resistant acid phosphatase-5b (TRACP-5b) concentrations were included as other outcomes. Supplemental Table 2 summarizes the assay methods, test kits, and measuring institution of these values. All biochemical samples were collected before the first HD session of the week (3 days after previous HD). Payne formula (in case of serum albumin [ALB] <4.0 g/dl, cCa=serum Ca+[4-ALB]) was used to calculate serum cCa.

### Safety Assessments

Clinical laboratory measurements, vital signs, and 12-lead electrocardiograms were examined for safety assessment (Supplemental Table 2). All adverse events, including those related to the upper gastrointestinal tract, were recorded and coded using MedDRA/J (version 21.1), and the preferred terms and system organ classes were used for tabulation. Investigators assessed the relationship between adverse events and the investigational drug. Symptomatic hypocalcemia was defined as “hypocalcemia.” Decreases in the serum Ca concentrations that were asymptomatic but judged by the investigator to be adverse events (regardless of the measured value) were recorded as “adjusted calcium decreased.”

### Statistical Analyses

Modified intent-to-treat (ITT) population, defined as ITT population excluding participants who never received any study drug or never underwent any efficacy assessments or measurements, was used to evaluate efficacy outcomes. The safety population was defined as all the participants who received the study drug at least once.

The sample size was established as follows: First, we chose an unbalanced randomized controlled trial design to minimize the number of participants assigned to the placebo group, with less clinical benefit. On the basis of our previous phase 2 study of upacicalcet, the percentage of participants achieving serum iPTH target concentrations in the upacicalcet group was conservatively assumed to be 45%. On the basis of a previous Japanese phase 3 study of cinacalcet, the proportion was assumed to be 10% for the placebo group.^6^ With a two-sided significance level of 5%, power of 90%, and upacicalcet-to-placebo ratio of 2:1, the required numbers were 56 and 28 in the upacicalcet and placebo groups, respectively. Regarding safety, upper gastrointestinal adverse events are known to be the major side effects of calcimimetics.^10^ The incidence of upper gastrointestinal adverse events was 3% (approximately one in 30) in our phase 2 study of upacicalcet. To detect the incidence of upper gastrointestinal adverse events with a sensitivity of ≥95%, 90 participants needed to be included in the upacicalcet group according to the rule of three. Therefore, a larger sample size was chosen between the two approaches, and the number of participants in this study was set to 150 (100 and 50 in the upacicalcet and placebo groups, respectively) considering early discontinuation of the study.

To analyze the primary and major secondary outcomes, the percentage of participants achieving target serum iPTH concentrations, ≥30% and ≥50% reductions between the groups were compared using the Fisher exact test. Participants who discontinued treatment had no measurements during the evaluation period or received rescue therapy with VDRAs (initiated or increased dose) were input as those who did not achieve these outcomes. Analyses of trends and changes in efficacy parameters were performed using the ITT principle, applying all available data, without imputation for missing data. Fisher exact test and two-way analysis of variance were used to compare the proportion and transition of each parameter between the groups. SAS (version 9.4; SAS Institute, Cary, NC) was used to perform statistical analyses and data review.

## Results

### Participant Disposition, Baseline Characteristics, and Dose

**Figure 1:**
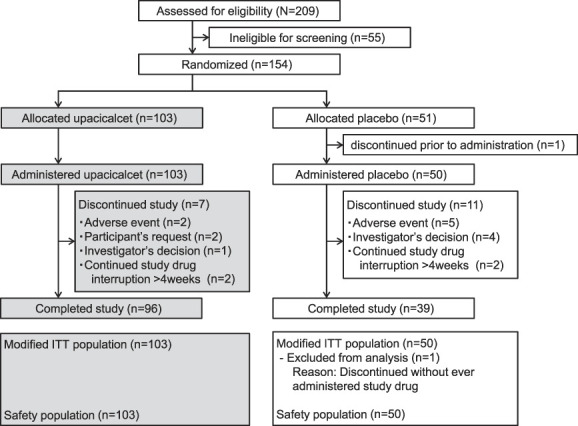
**Participant flow.** ITT, intent-to-treat.

A total of 154 participants were enrolled and randomized into either an upacicalcet or placebo treatment group. Among the participants included, one participant in the placebo group withdrew from the study before administration owing to high alanine aminotransferase and aspartate aminotransferase concentrations caused by choledocholithiasis; the remaining participants were administered the respective study drugs. The backgrounds of the participants in the two groups were similar (Table 1). Nearly half of the participants were non-calcimimetic users. The mean duration of dialysis for the participants was approximately 10 years. Approximately 30% of participants were treated with a dialysate calcium concentration of 3.0 mEq/L. Of these, 93% and 78% of participants in the upacicalcet and placebo groups, respectively, completed the 24-week study. (Figure 1). The administration rate of the study drug was >95% throughout the study period in both groups (Supplemental Figure 2).

**Table 1:** Baseline characteristics of participants in a randomized clinical trial of upacicalcet in Japan

Participant Characteristics	Classification	Upacicalcet (*n*=103)	Placebo (*n*=50)
Sex, *n* (%)	Male	77 (75)	39 (78)
	Female	26 (25)	11 (22)
Age, yr	Mean±SD	62±13	66±14
	≥65	55 (53)	30 (60)
Primary disease, *n* (%)	Chronic glomerulonephritis	36 (35)	24 (48)
	Diabetic kidney disease	33 (32)	10 (20)
	Nephrosclerosis	13 (13)	5 (10)
	Polycystic kidney disease	2 (2)	1 (2)
	Other, including unknown	19 (18)	10 (20)
Duration of dialysis, yr, *n* (%)	Mean±SD	10±8	8±7
	<5	35 (34)	25 (50)
	5–9	21 (20)	6 (12)
	10–19	32 (31)	16 (32)
	≥20	15 (15)	3 (6)
Dialysate calcium concentration, mEq/L, *n* (%)	2.5	32 (31)	14 (28)
	2.75	39 (38)	21 (42)
	3.0	32 (31)	15 (30)
Use of vitamin D receptor activators, *n* (%)	No	27 (26)	7 (14)
	Yes	76 (74)	43 (86)
Use of calcium carbonate, *n* (%)	No	56 (54)	29 (58)
	Yes	47 (46)	21 (42)
Use of non–calcium-containing phosphate binders, *n* (%)	No	15 (15)	10 (20)
	Yes	88 (85)	40 (80)
Prior use of calcimimetics, *n* (%)	Cinacalcet	16 (16)	12 (24)
	Etelcalcetide	3 (3)	2 (4)
	Evocalcet	39 (38)	13 (26)
	None	45 (44)	23 (46)
Serum intact parathyroid hormone, pg/ml	Median (Q1, Q3)	364 (306, 463)	369.5 (302, 457)
Category of serum intact parathyroid hormone, pg/ml	≤240	5 (5)	4 (8)
	241–499	79 (77)	38 (76)
	≥500	19 (18)	8 (16)
Serum corrected calcium, mg/dl	Mean±SD	9.3±0.7	9.4±0.7
Category of serum corrected calcium, mg/dl, *n* (%)	<8.4	2 (2)	2 (4)
	8.4–8.9	37 (36)	15 (30)
	9.0–10.0	49 (48)	23 (46)
	>10.0	15 (15)	10 (20)
Serum phosphate, mg/dl	Mean±SD	6.0±1.4	6.2±1.6
Serum fibroblast growth factor 23, pg/ml	Median (Q1, Q3)	8670 (2080, 18,900)	8485 (3700, 28,700)
Serum bone alkaline phosphatase, *µ*g/L	Median (Q1, Q3)	15.7 (12.1, 20.9)	15.5 (11.6, 20.5)
Serum N-terminal propeptide of type 1 procollagen, ng/ml	Median (Q1, Q3)	356 (243, 493)	330.5 (205, 472)
Serum tartrate-resistant acid phosphatase-5b, mU/dl	Median (Q1, Q3)	723 (495, 963)	717 (415, 980)

Data are shown as number (percentage), mean±SD, or median (25%tile, 75%tile).

### Efficacy Evaluations

#### Primary Outcome Measure

**Figure 2:**
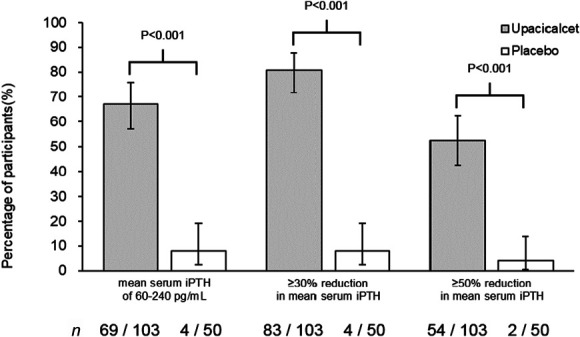
**Percentage of participants achieving primary and major secondary outcomes.** Data are shown as percentages and their 95% confidence intervals. Fisher exact test was used for comparisons between groups. *n* denotes achieving participants/overall participants (modified intent-to-treat population). iPTH, intact parathyroid hormone.

The percentage of participants achieving mean serum iPTH concentrations of 60–240 pg/ml during the evaluation period was 67% (95% confidence interval [CI], 57% to 76%) in the upacicalcet group and 8.0% (95% CI, 2% to 20%) in the placebo group. The difference in percentage between the two groups was 59% (95% CI, 48% to 71%), which was statistically significant (*P* < 0.001) (Figure 2, Supplemental Figure 3). The reasons why the primary outcome was not achieved are presented in Supplemental Table 3.

#### Secondary Outcome Measure

The percentages of participants achieving ≥30% and ≥50% reductions in mean serum iPTH concentrations from baseline during the evaluation period were 81% (95% CI, 72% to 88%) and 52% (95% CI, 42% to 62%) in the upacicalcet group and 8.0% (95% CI, 2% to 20%) and 4% (95% CI, 1% to 14%) in the placebo group, respectively. The differences in percentage between the two groups were 73% (95% CI, 62% to 83%) and 49% (95% CI, 37% to 60%), respectively, which were statistically significant (*P* < 0.001) (Figure 2, Supplemental Figure 3).

**Figure 3:**
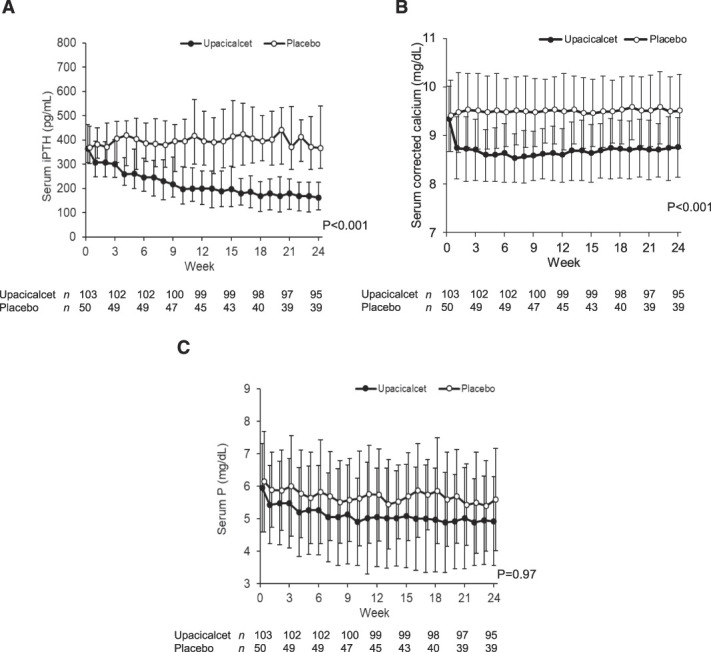
**Changes in serum iPTH, serum corrected calcium, and serum P concentrations.** (A) Serum iPTH concentrations over time. (B)Serum corrected calcium concentrations over time. (C) Serum P concentrations over time. Data are shown as median with interquartile range (A) and mean±SD (B, C). Two-way analysis of variance was used for comparisons between groups.

The median (interquartile range) serum iPTH concentrations at weeks 0 and 24 were 364 (306–463) and 161 (111–225) pg/ml in the upacicalcet group and 369.5 (302–457) and 367 (282–540) pg/ml in the placebo group, respectively. The difference in change between the two groups was statistically significant (*P* < 0.001) (Figure 3A). All subgroup analyses of serum iPTH and cCa concentrations, VDRA use, dialysis history, and dialysate Ca concentration suggested that upacicalcet lowered serum iPTH concentrations (Table 2, Supplemental Table 4).

**Table 2:** Subgroup analysis of percent changes in serum intact parathyroid hormone concentrations at week 24

Percent Change of Serum iPTH Concentration	Upacicalcet		Placebo		Difference between Two Groups	*P* Value for Interaction
	No. of Participants	Percent Change (%)	No. of Participants	Percent Change (%)		
Overall participants	95	−53±23	39	8±38	−61 (−74 to −48)	
**Subgroups of baseline serum iPTH concentrations, pg/ml**						0.03
≥500	18	−59±26	6	16±27	−74 (−100 to −49)	
241–499	73	−53±22	31	2±37	−55 (−69 to −40)	
≤240	4	−44±17	2	69±47	−113 (−180 to −46)	
**Subgroups of baseline serum corrected calcium concentrations, mg/dl**						0.10
<8.4	2	−31±58	2	1±9	−33 (−210 to 145)	
8.4–8.9	33	−55±26	12	−9±34	−46 (−65 to −27)	
9.0–10.0	47	−54±20	18	11±37	−65 (−84 to −45)	
>10.0	13	−50±18	7	30±45	−81 (−123 to −39)	
**Subgroups of baseline VDRA use**						0.10
User of VDRA	73	−56±21	35	9±40	−65 (−80 to −51)	
Nonuser of VDRA	22	−43±26	4	−4±12	−39 (−67 to −12)	
**Subgroups of duration of dialysis, yr**						0.36
<5	33	−57±24	21	5±43	−62 (−83 to −42)	
5–9	18	−50±16	4	−6±10	−45 (−63 to −27)	
10–19	31	−54±25	11	19±41	−73 (−101 to −45)	
≥20	13	−46±22	3	1±9	−47 (−75 to −19)	
**Subgroups of dialysate calcium concentration, mEq/L**						0.02
2.5	32	−50±25	13	−7±37	−42 (−62 to −23)	
2.75	35	−59±23	16	7±35	−66 (−83 to −50)	
3.0	28	−50±18	10	27±38	−78 (−97 to −59)	

Data are shown as mean±SD. Differences between groups are shown as point estimates and 95% confidence intervals. iPTH, intact parathyroid hormone; VDRA, vitamin D receptor activator.

The mean serum cCa concentrations at weeks 0 and 24 were 9.3±0.7 and 8.8±0.6 mg/dl in the upacicalcet group and 9.4±0.7 and 9.5±0.8 mg/dl in the placebo group, respectively. The difference in change between the two groups was statistically significant (*P* < 0.001) (Figure 3B).

The mean serum P concentrations at weeks 0 and 24 were 6.0±1.4 and 5.0±1.4 mg/dl in the upacicalcet group and 6.2±1.6 and 5.6±1.6 mg/dl in the placebo group, respectively (Figure 3C).

#### Other Outcome Measures

**Figure 4:**
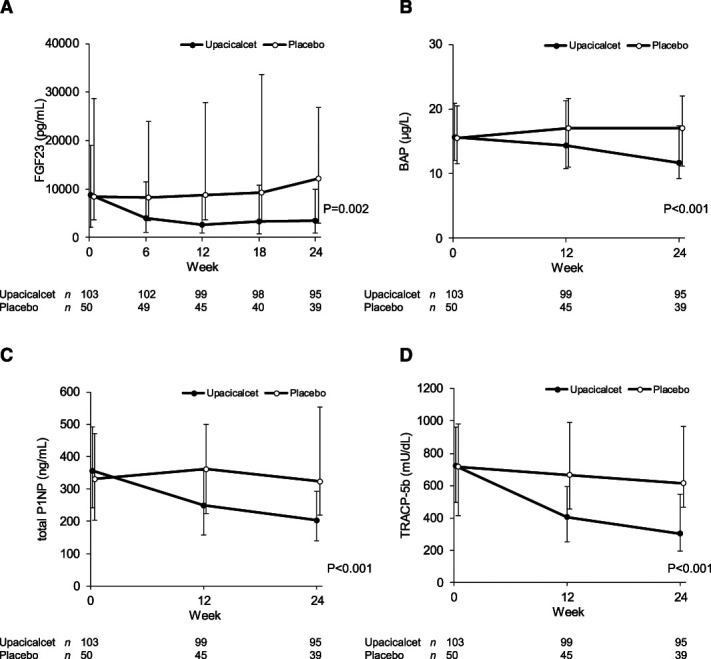
**Changes in FGF23 and bone metabolic markers as other outcomes.** (A) Serum FGF23 concentrations over time. (B) Serum BAP concentrations over time. (C) Serum total P1NP concentrations over time. (D) Serum total P1NP concentrations over time. Data are shown as median with interquartile range. Two-way analysis of variance was used for comparisons between groups. BAP, bone alkaline phosphatase; FGF23, fibroblast growth factor 23; P1NP, N-terminal propeptide of type 1 procollagen; TRACP-5b, tartrate-resistant acid phosphatase-5b.

The differences in the changes in FGF23, BAP, total P1NP, and TRACP-5b concentrations between the upacicalcet and placebo groups were statistically significant (Figure 4).

### Safety Evaluations

Adverse events were reported in 85% (88/103) and 72% (36/50) of participants in the upacicalcet and placebo groups, respectively (*P* = 0.076) (Table 3). Investigational drug-related adverse events were reported in 12% of the participants in the upacicalcet group and 8% of those in the placebo group (*P* = 0.583). One death due to bacterial meningitis was reported in the upacicalcet group, but it was considered to be unrelated to the study drug by the investigator. Investigational drug-related adverse events reported in the upacicalcet group were adjusted calcium decreased in nine participants (9%) and nausea, shunt thrombosis, decreased appetite, and muscle spasms in one participant (1%). These were considered nonserious events.

**Table 3:** Summary of adverse events

Adverse Events	Adverse Events		Investigational Drug-Related Adverse Events	
	Upacicalcet (*n*=103)	Placebo (*n*=50)	Upacicalcet (*n*=103)	Placebo (*n*=50)
Any adverse events, *n* (%)	88 (85)	36 (72)	12 (12)	4 (8)
Death, *n* (%)	1 (1)	0 (0)	0 (0)	0 (0)
Serious adverse events not including death, *n* (%)	11 (11)	8 (16)	0 (0)	1 (2)
**Upper gastrointestinal symptoms, *n* (%)**	21 (20)	9 (18)	2 (2)	3 (6)
Vomiting	5 (5)	4 (8)	0 (0)	1 (2)
Nausea	3 (3)	1 (2)	1 (1)	0 (0)
Abdominal discomfort	2 (2)	3 (6)	0 (0)	1 (2)
Abdominal distension	1 (1)	0 (0)	0 (0)	0 (0)
Decreased appetite	3 (3)	1 (2)	1 (1)	0 (0)
Hypocalcemia, *n* (%)	0 (0.0)	0 (0)	0 (0.0)	0 (0)
Adjusted calcium decreased, *n* (%)	9 (9)	0 (0)	9 (9)	0 (0)

Data are shown as number (percentage).

Upper gastrointestinal adverse events were reported in 20% of participants in the upacicalcet group and 18% of those in the placebo group (*P* = 0.829). The incidence of each symptom was also not significantly different between the two groups (Table 3).

Symptomatic hypocalcemia was not reported in either groups. Investigational drug-related adverse event adjusted calcium decreased was reported in 9% of the participants in the upacicalcet group. Two participants in the upacicalcet group each had serum cCa concentrations <7.5 mg/dl once (Supplemental Table 6) and were interrupted on study drug administration. One of these participants increased the dose of calcium carbonate. Their serum cCa concentrations recovered to >8.4 mg/dl a week. Both participants resumed the same dose of upacicalcet as before interruption, and their corrected Ca concentration did not drop to <7.5 mg/dl until the end of the study. A trend of increased calcium carbonate dosage was observed in the upacicalcet group (Supplemental Table 7). In the 12-lead electrocardiograms, the change in QT corrected by Fridericia's formula from weeks 0 to 24 was 7.1±15.7 ms (90% CI, 4.5 to 9.8 ms) in the upacicalcet group and 0.8±16.7 ms (90% CI, −3.7 to 5.3 ms) in the placebo group. Adverse events associated with QT prolongation were not reported in either of the groups.

## Discussion

In this study, upacicalcet therapy achieved the serum iPTH target concentration (60–240 pg/ml) on the basis of the CKD–MBD guidelines by JSDT at a 59% higher proportion than in placebo therapy.^21^ This result is similar to that observed for etelcalcetide treatment in Japan, which achieved a 60% higher iPTH target concentration than the placebo.^22^ On the basis of these findings, upacicalcet may be comparable with etelcalcetide in achieving the serum iPTH target concentration. However, a direct comparative study of these two injectable calcimimetics is required to clarify the noninferiority. Subgroup analysis suggested that upacicalcet can be effective in diverse patients with secondary hyperparathyroidism. However, because the target serum iPTH concentration recommended by the JSDT guidelines is lower than that recommended in the Kidney Disease Improving Global Outcomes guidelines,^4,21^ the serum iPTH concentrations of HD patients in this Japanese study may be lower than those of patients from other countries. Therefore, further studies are needed to evaluate whether ≤300 *μ*g of upacicalcet is effective in patients with extremely high serum iPTH concentrations.

Regarding bone metabolic markers, upacicalcet significantly decreased the serum BAP, total P1NP, and TRACP-5b concentrations. Patients with secondary hyperparathyroidism present with high-turnover bone disease and decreased cortical bone mass.^23^ Upacicalcet lowering bone metabolic marker concentrations besides lowering serum iPTH concentration may positively affect bone structure. Further studies confirming this hypothesis are required.

Upacicalcet significantly decreased the serum FGF23 concentrations. Elevated FGF23 concentrations are associated with cardiovascular events, infections, and death.^24–27^ Cinacalcet treatment was reported to reduce serum FGF23 concentrations, which was associated with the prevention of cardiovascular mortality and heart failure.^28^ In our study, the median changes in serum FGF23 after 24 weeks of upacicalcet and placebo treatment were 60% and 3%, respectively. Thus, upacicalcet might prevent cardiovascular mortality and heart failure by decreasing serum FGF23 concentrations, like cinacalcet. However, further long-term studies are required to confirm this hypothesis.

Regarding safety evaluations, the major adverse events associated with calcimimetics are upper gastrointestinal symptoms and hypocalcemia. Cinacalcet is known to induce upper gastrointestinal disturbances, leading to low adherence.^10,11^ Evocalcet, a new oral calcimimetic, was reported to have a higher bioavailability, reduced gastrointestinal exposure, and thus, fewer incidences of upper gastrointestinal disturbances than cinacalcet.^13,29^ Intravenous calcimimetics, such as etelcalcetide, were reported to cause nausea and vomiting more commonly than placebo and at a similar frequency to cinacalcet, against expectation.^15–17^ On the contrary, upacicalcet did not increase the incidence of complaints of nausea and vomiting compared with placebo in this study. From a pharmacologic viewpoint, it is reasonable to assume that upacicalcet causes gastrointestinal disturbances; concordantly, we found that upacicalcet doses >400 *µ*g could cause upper gastrointestinal adverse events.^20^ However, here we showed that upacicalcet with dose titration up to 300 *µ*g could reduce serum iPTH concentrations in participants with mild-to-severe (serum iPTH ≥500 pg/ml) secondary hyperparathyroidism on HD while suppressing gastrointestinal adverse events. Widespread upacicalcet use in the real world should confirm the exact incidence of upper gastrointestinal adverse events, tolerability, and adherence of it.

Whether hypocalcemia due to calcimimetics is acceptable remains a debatable issue.^4,30^ However, severe or prolonged hypocalcemia is a risk factor of QT prolongation, arrhythmia, and death.^31–33^ Upacicalcet reduced serum cCa concentrations, similar to other calcimimetics, and thus promoted calcium carbonate increment. Nevertheless, only 2% of the participants had serum cCa concentrations <7.5 mg/dl, the threshold for the interruption of calcimimetics according to the drug labels in Japan. Moreover, the hypocalcimetic state of these participants recovered a week after interrupting upacicalcet administration. Furthermore, upacicalcet did not cause symptomatic hypocalcemia. This uncommon/rapidly subsiding hypocalcemia can be supported by the pharmacokinetic properties of upacicalcet. Upacicalcet's interdialytic half-life of plasma and removal rate in a HD session ranged between 65 and 122 hours and 79% and 100% in HD patients, respectively.^20^ Thus, the plasma upacicalcet concentration plateaus after repeated 1-week dosing.^20^ The pharmacokinetic properties of upacicalcet differ from those of etelcalcetide, which forms a serum albumin peptide conjugate and accumulates in the plasma.^34^ A careful dosing titration regimen of upacicalcet may lower serum iPTH concentrations, avoiding hypocalcemia associated with excessive drug effects.

This study has several limitations. First, only Japanese patients receiving HD were included. In Japan, compared with other countries, serum iPTH is controlled at low concentrations, and dialysate with a Ca concentration of 3.0 mEq/L is widely used. Further studies on other ethnic groups and populations undergoing HD are required to generalize the efficacy and safety of upacicalcet. Second, the intervention period in this study was only 24 weeks. The study was designed to verify the superiority of upacicalcet over placebo, with the primary outcome emphasizing a reduction in serum PTH concentrations. The further long-term trial result to assess durability of effect and safety through sustained exposure of upacicalcet will be reported soon. Third, the upper gastrointestinal adverse events in this study were not prospectively ascertained, but were recorded as complained of by the participants. More detailed prospective studies are warranted to clarify whether upacicalcet does not cause upper gastrointestinal disturbances.

In conclusion, upacicalcet significantly reduced serum iPTH concentrations, serum FGF23 concentrations, and bone metabolism marker concentrations. Upacicalcet-induced gastrointestinal disturbances and hypocalcemia were uncommon. Thus, upacicalcet is effective and can be a safe calcimimetic agent for the management of secondary hyperparathyroidism.

## Supplementary Material

## Supplemental Material

Supplemental Document 1. List of exclusion criteria.

Supplemental Figure 1. Study design.

Supplemental Figure 2. Time course of changes in study drug dosing in the upacicalcet group (A) and placebo group (B).

Supplemental Figure 3. Percentage of participants achieving primary and major secondary outcomes using multiple imputation methods (*ad hoc* analyses).

Supplemental Table 1. List of participating institutions and investigators.

Supplemental Table 2. Schedule of screening, treatment, and assessment.

Supplemental Table 3. List of reasons why the primary outcome was not achieved.

Supplemental Table 4. Subgroup analysis of percent changes in the serum iPTH concentrations at week 24 using mixed-effects models for repeated measures (*ad hoc* analyses).

Supplemental Table 5. List of all adverse events during the treatment period.

Supplemental Table 6. Distribution of the lowest serum corrected calcium concentrations during the treatment period.

Supplemental Table 7. Summary of concomitant phosphate binders and vitamin D receptor activators over time.
