# Supplementary figures and images for: Efficacy and Safety of Upacicalcet in Hemodialysis Patients with Secondary Hyperparathyroidism: A Randomized Placebo-Controlled Trial
Source: Clin J Am Soc Nephrol. 2023 Sep 11;18(10):1300–9. doi: 10.2215/CJN.0000000000000253 (PMC10578632; doi:10.2215/CJN.0000000000000253)

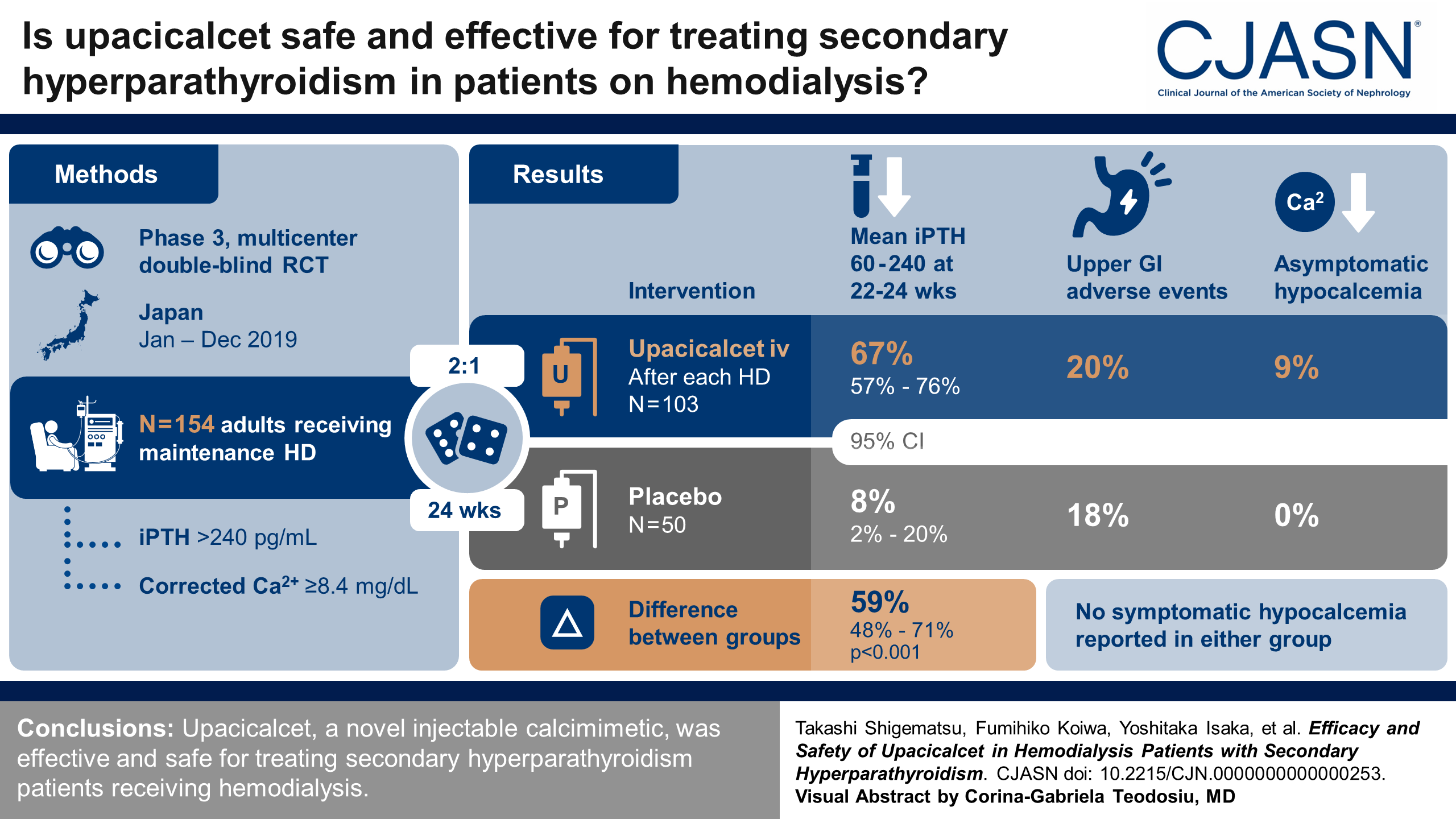

Supplement: Supplementary file 1 [file cjasn-18-1300-s001.png]
